# Monocytes promote acute neuroinflammation and become pathological microglia in neonatal hypoxic-ischemic brain injury

**DOI:** 10.7150/thno.64033

**Published:** 2022-01-01

**Authors:** Hong-Ru Chen, Ching-Wen Chen, Yi-Min Kuo, Brandon Chen, Irena S. Kuan, Henry Huang, Jolly Lee, Neil Anthony, Chia-Yi Kuan, Yu-Yo Sun

**Affiliations:** 1 Department of Neuroscience, Center for Brain Immunology and Glia (BIG), University of Virginia School of Medicine, Charlottesville, VA, USA.; 2 Department of Anesthesiology, Taipei Veterans General Hospital and National Yang Ming Chiao Tung University School of Medicine, Taipei, Taiwan.; 3 University of Louisville School of Medicine, Louisville, KY, USA.; 4 St. Louis University School of Medicine, St. Louis, MO, USA.; 5 Department of Anesthesiology, Rhode Island Hospital, Providence, RI, USA.; 6 Emory University School of Medicine, Atlanta, GA, USA.; 7 Emory Integrated Cellular Imaging, Atlanta, GA, USA.; 8 Institute of BioPharmaceutical Sciences, National Sun Yat-sen University, Kaohsiung, Taiwan.

**Keywords:** CCR2, microglia, chorioamnionitis, hypoxic ischemic encephalopathy (HIE), monocyte-derived macrophages, neuroinflammation

## Abstract

**Rationale:** Monocytes belong to the mononuclear phagocyte system and are immune responders to tissue injury and infection. There were also reports of monocytes transforming to microglia-like cells. Here we explore the roles of monocytes in microglia ontogeny and the pathogenesis of neonatal cerebral hypoxic-ischemic (HI) brain injury in mice.

**Methods:** We used three genetic methods to track the development of monocytes, including CX3CR1^GFP/+^; CCR2^RFP/+^ reporter mice, adoptive transfer of GFP^+^ monocytes, and fate-mapping with CCR2-CreER mice, in neonatal mouse brains with or without lipopolysaccharide (LPS, 0.3 mg/kg)-sensitized Vannucci HI. We also used genetic (CCR2^RFP/ RFP^, CCR2 knockout) and pharmacological methods (RS102895, a CCR2 antagonist) to test the roles of monocytic influx in LPS/HI brain injury.

**Results:** CCR2^+^ monocytes entered the late-embryonic brains via choroid plexus, but rapidly became CX3CR1^+^ amoeboid microglial cells (AMCs). The influx of CCR2^+^ monocytes declined after birth, but recurred after HI or LPS-sensitized HI (LPS/HI) brain injury, particularly in the hippocampus. The CCR2-CreER-based fate-mapping showed that CCR2^+^ monocytes became CD68^+^ TNFα^+^ macrophages within 4 d after LPS/HI, and maintained as TNFα^+^ MHCII^+^ macrophages or persisted as Tmem119^+^ Sall1^+^ P2RY12^+^ ramified microglia for at least five months after injury. Genetic deletion of the chemokine receptor CCR2 markedly diminished monocytic influx, the expression of pro- and anti-inflammatory cytokines, and brain damage. Post-LPS/HI application of RS102895 also reduced inflammatory responses and brain damage, leading to better cognitive functions.

**Conclusion:** These results suggest that monocytes promote acute inflammatory responses and may become pathological microglia long after the neonatal LPS/HI insult. Further, blocking the influx of monocytes may be a potential therapy for neonatal brain injury.

## Introduction

Inflammation is an important risk factor for neonatal brain injury, since asphyxiated infants with intrauterine infection (chorioamnionitis) have poorer neurological outcomes even after hypothermia treatment 1, 2. In inflammation-sensitized neonatal HI injury, the influx of lymphocytes and neutrophils worsens brain damage, but the functions of monocytes remain uncertain 3-6. It has been reported that the resident microglia, but not monocyte-derived macrophages, contribute to faster and more severe inflammatory responses to neonatal HI brain injury 7. This is surprising, since monocytes have been shown to promote pathology in many experimental models, including neonatal stroke 8, 9. Alternatively, monocytic infiltrates may be more important for inflammation-sensitized neonatal HI brain injury, but this scenario is yet to be tested.

Murine monocytes consist of a long-living CCR2^-^ CX3CR1^+^ Ly6C^lo^ patrolling subtype and a short-lived CCR2^+^CX3CR1^-^ Ly6C^hi^ pro-inflammatory subtype that invade the damaged tissue 10-12. The CCR2^RFP/+^ knock-in mice is a great tool to detect monocytes, especially when they were crossed with CX3CR1^GFP/+^ mice that label the resident microglia with GFP 13, 14. Through the use of parabiosis and CCR2^RFP/+^; CX3CR1^GFP/+^ reporter mice, it has been shown that the brain resident microglia are sustained by self-renewal, while Ly6C^hi^ monocytes only enter the brain parenchyma after injury with apparent blood-brain-barrier (BBB) damage 15, 16. Further, the fates of monocytic infiltrates may depend on the type of brain insults. In experimental autoimmune encephalitis (EAE), monocytic infiltrates promote acute brain pathology, but do not contribute to the resident microglia pool 17. In contrast, the monocytic infiltrates were reported to survive longer after status epilepticus, peripheral nerve injury, and cerebral ischemia 18-21. Interestingly, it has been reported that monocytes undergo *in-situ* transition from a CCR2^hi^ CX3CR1^low^ to CCR2^lo^ CX3CR1^hi^ state after sterile liver injury 22. These findings suggest that the combination of ontogeny and environment may determine the final fate of monocytic infiltrates after brain injury 23-25.

Whether monocytes contribute to the brain microglia pool in development is another contentious issue over the past century 26, 27. The classical view held that microglia derive from the mononuclear phagocyte system from late embryogenesis to the neonatal period 27, 28. However, research in the past decade has challenged this view and revealed that mouse microglia originate from the yolk sac-derived myeloid progenitors around embryonic day 7.5 (E7.5) and migrate into the cephalic mesenchyme at E10.5 to expand perinatally with little or no inputs by peripheral monocytes that descend from fetal hematopoietic stem cells 29, 30. Nevertheless, two recent studies suggested considerable monocyte-to-microglia transition in development 9, 31. These new findings seemingly contradict a previous report that CCR2^+^ monocytes were confined between the surface ectoderm and neuroepithelium in CCR2^RFP/+^; CX3CR1^GFP/+^ embryos 32. One scenario to reconcile these findings is that monocytes may enter the developing brain through specific “microglial fountains” locations 33, and rapidly change their cell surface markers from a CCR2^hi^ CX3CR1^low^ to CCR2^lo^ CX3CR1^hi^ state.

To test this hypothesis, here we re-examined CCR2^RFP/+^; CX3CR1^GFP/+^ embryos, and used adoptive transfer of CCR2^RFP/+^; actin-GFP^+^ monocytes plus tamoxifen-induced CCR2-CreER; R26R-EGFP mice to assess the fate of monocytes after neonatal LPS/HI injury. Our results suggest significant plasticity of monocytes and long-term adverse effects in neonatal HI brain injury. Moreover, both genetic and pharmacological blockade of monocytic influx diminished neonatal LPS/HI brain damage. Together, these findings suggest that monocytic infiltration may be a promising therapeutic target in inflammation-sensitized neonatal brain injury.

## Materials and Methods

### Mice

The wild-type C57BL/6J breeders were purchased from Charles River Laboratories. CCR2^RFP/RFP^ mice (JAX #017586) and CX3CR1^GFP/GFP^ (JAX #005582) mice were purchased from the Jackson Laboratory. CCR2^RFP/RFP^ and CX3CR1^GFP/GFP^ mice were crossed to obtain CCR2^RFP/+^ CX3CR1^GFP/+^ pups for most experimental purposes. CCRR2^RFP/+^; Actin-GFP mice were derived from CCR2^RFP/RFP^ and Actin-GFP mice (JAX #003291) for adoptive transfer of monocytes. CCR2-CreER(T2) mice have been characterized 9. The CCR2-CreER mice were crossed with a R26R-GFP Cre-reporter line (Ai6, JAX #007906) or R26R-GFP/Rpl10A mice (JAX #022367). For fate-mapping, the progeny of CCR2-CreER crossed to R26R-GFP or R26R-GFP/Rpl10A mice received 10 mg/kg/day tamoxifen (#T5648, Sigma-Aldrich) at P8 and P9, followed by HI surgery at P10.

### Animal Surgery and treatment

The LPS-sensitized hypoxic-ischemic (HI) injury was performed as previously described 4, 34. Briefly, LPS (0.3 mg/kg) was injected intraperitoneally to P10 pups at 4 h prior to the Vannucci HI procedure with the right common carotid artery ligation followed by 10% hypoxia for 40 min. Those pups were randomly selected for treatment with two doses of RS102895 (5 to 20 mg/kg #R1903, Sigma-Aldrich) or vehicle [20% (2-Hydroxypropyl)-β-cyclodextrin solution, #H107, Sigma-Aldrich] immediately after hypoxia and at 1 h recovery. Both male and female pups were examined and showed no discernible differences in response. Surgical procedures were approved by the Institutional Animal Care and Use Committee (IACUC) and conform to the NIH Guide for Care and Use of Laboratory Animals.

### Histological assay of microglia distribution and monocytic infiltration

CCR2^RFP/+^; CX3CR1^GFP/+^, CCR2^RFP/RFP^ or CCR2^RFP/+^ pups were subjected to transcardial perfusion of phosphate-balanced saline (PBS) and 4% paraformaldehyde. The fixed brains were dehydrated by 30% sucrose and frozen in optimal cutting-temperature (OCT) compound for 20 μm sections. GFP^+^ microglia and RFP^+^ monocyte were observed on an epifluorescence microscope (BX43, Olympus) or a confocal microscope (SP8 Leica). Images were randomly selected in 5 visual fields with 20X objective and RFP^+^ cells were quantified using ImageJ software (NIH).

### Live brain slice imaging

The embryonic brains were collected from timed female CCR2^RFP/+^ CX3CR1^GFP/+^ mice at day 17 of gestation. The brains were embedded in 4% low melting point agarose (Sigma), and cut into 300 μm coronal section by a vibrating microtome (Electron Microscopy Sciences). The brain slices were transferred to a glass bottom imaging dish (Ibidi) and covered by a drop of Matrigel (BD Biosciences). The brain slices were incubated in DMEM/F-12 with 5% fetal bovine serum. GFP+ and RFP+ cells were imaged by a confocal microscope with on-stage incubator in 5% CO2 and 37 °C environment (SP8 Leica).

### Adoptive transfer

P10 C57BL/6 wild-type mice subjected to LPS/ HI insult were intravenously injected via retro-orbital with 3 × 10^5^ CCR2^RFP/+^ Actin-GFP^+^ bone marrow monocytes purified using the monocyte isolation kit (#130-100-629, Miltenyi Biotec). The brains collected at 1, 3, and 14 d after adoptive transfer were performed with cryosection procedures followed by anti-GFP or the stated staining.

### Immunohistochemistry and immunoblotting analysis

Immunohistochemistry and immunoblotting were performed as previously described 4, 34. The following antibodies for immunohistochemistry were used: rabbit anti-GFP (#A6455, Invitrogen), rabbit anti-MCP-1 (#500-P113, PeproTech), mouse anti-TNFα (#ab8348, Abcam), rabbit anti-NeuN (#ab177487, Abcam), rabbit anti-Iba1 (#019-19741, Wako), rabbit anti-Ki67 (#ab15580, Abcam), rat anti-CD68 (#FA-11, Abcam), rabbit anti-Tmem119 (#ab209064, Abcam), rat anti-P2RY12 (#848001, BioLegend), rabbit anti-Synaptotagmin 1 (#105003, Synaptic Systems), mouse anti-MHC-II (#ab55152, Abcam), and mouse anti-PSD-95 (#MAB1596, EMD Millipore). Species-specific secondary antibodies were conjugated with Alexa Fluor488, 594 or 647 (Molecular Probes, Invitrogen). The antibodies for immunoblotting were: rabbit anti-iNOS (#610332, BD PharMingen), mouse anti-COX2 (#610204, BD PharMingen), and mouse anti-GAPDH (#MAB374, EMD Millipore).

### Cell proliferation assay

The 5-ethynyl-2'-deoxyuridine (EdU, #900584, Sigma-Aldrich) was injected (10mg/kg, i.p.) at 2 h prior to brain collection for immunostaining using a commercial kit (Click-iT^TM^ Plus EdU cell proliferation kit, Invitrogen).

### Brain immune cell isolation and flow cytometry

Infiltrated immune cells in neonatal brains for flow cytometry were performed as described 4. Briefly, gently homogenized brain tissues were laid on discontinued Percoll gradients and the infiltrating immune cells in 70%-30% Percoll (GE Healthcare) interphase were collected. The isolated immune cells were stained with antibodies described as below, anti-CD45 (clone 30-F11, BioLegend), anti-CD11b (clone M1/ 70, BD PharMingen), anti-Ly6G (clone 1A8, BioLegend), anti-Ly6C (clone HK1.4), anti-IL1b (clone NJTEN3, eBioscience), LIVE/DEAD Fixable Dead Cell Stain kit (Invitrogen). Isolated cells were incubated with PMA and ionomycin in the presence of GolgiPlug/ GlogiStop for 5 h, the intracellular cytokines staining was performed after fixation and permeabilization (Cytofix/Cytoperm^TM^, BD Biosciences). The absolute cell counts were obtained from CountBright counting beads (Invitrogen). Data was acquired on an Attune NxT flow cytometer (Invitrogen) or Imagestream X Mark II Imaging Cytometer (Luminex). Spectral overlap was compensated, and data analyzed using FlowJo software (Treestar Inc).

### Microglia culture

Immortalized primary mouse microglial in Balb/c background (SM826 cells) were cultured in 10% FBS/ DMEM medium (Invitrogen) 4. The CCR2 inhibitor RS102895 (Sigma-Aldrich) was added at 30 min prior to LPS incubation (#5418, Sigma-Aldrich), followed by mRNA and protein analysis 24 h later.

### Apoptosis assay

Cell apoptosis was performed via a commercial kit (Click-iT^TM^ Plus TUNEL assay, Invitrogen). TUNEL^+^ cells were detected at an emission of 647 nm and quantified as the ratio to DAPI-positive nuclei in five randomly selected visual fields.

### Quantitative real-time PCR

The total RNA was isolated from neonatal brain tissues and microglia cells using the TRIzol® RNA isolation kit (#15596-026, Invitrogen). Quantitative real-time PCR was performed using a Bio-Rad CFX 96 system (C1000 Thermal Cycler, Bio-Rad) and detected by SYBR Green master mix (Bio-Rad) as previous described 4. The following primer sequences were used for PCR.

- *Tnf* CCACCACGCTCTTCTGTCTA; CTCCTCCACTTGGTGGTTTG;
- *Il1b* CTTTCGACAGTGAGGAGAATGAC; CAAGACATAGGTAGCTGCCACAG;
- *Il23a* ACCAGTGGGACAAATGGATCTAC; CAGGTGCTTATAAAACACCAGACC;
- *Il6* GGAGAGGAGACTTCACAGAGGAT; AGTGCATCATCGCTGTTCATAC;
- *Il10* AAGGACCAGCTGGACAACAT; TCCTGAGGGTCTTCAGCTTC;
- *Arg1* CGCCTTTCTCAAAAGGACAG; CCAGCTCTTCATTGGCTTTC;
- *Chil3/* Ym1 ACCAGTTGGGCTAAGGACAG; TGGCCAGGAGAGTTTTTAGC;
- *Ccl2/* Mcp1 ACCACTATGCAGGTCTCTGTCAC; GCTGCTGGTGATTCTCTTGTAGT;
- *Nos2/* iNOS TACCAAAGTGACCTGAAAGAGGA; ATTCTGGAACATTCTGTGCTGTC;
- *Tspo* CTATGGTTCCCTTGGGTCTCTAC; AGGCCAGGTAAGGATACAGCAAG;
- *Actb/* β-actin GGCACCACACTTTCTACAATGA; AGTGGTACGACCAGAGGCATAC;
- *Gapdh* CTCATGACCACAGTCCATGC; TTCAGCTCTGGGATGACCTT.

### Brain atrophy measurement

Brain atrophy was performed using Nissl stain (cresyl violet, Sigma-Aldrich) at 7 d after neonatal LPS/HI insults by a lab member blinded to the mouse genotype and treatment. Fixed brains were sectioned to 1 mm thickness slices (6 per brain) and analyzed using the ImageJ 1.4 software (NIH). The percentage of tissue loss in the cerebral cortex and hippocampus was quantified as the ratio of the stated ipsilateral to the contralateral area.

### Novel objective recognition test

Behavior assessment of hippocampal-related novel object recognition (NOR) test was performed in vehicle or CCR2 antagonist injected mice by an experimenter blinded to the treatments. On 2 d prior to the NOR testing, mice were allowed to explore the empty NOR apparatus (40 cm L; 40 cm W; 40 cm H) freely for 10 min. On 1 d prior to the NOR testing, mice were given an exploration period in the apparatus with two identical (familiar) objects, one in the upper right and the other in the lower left quadrants of the apparatus for 10 min. On the day of NOR testing, each mouse was placed in the apparatus and exposed to two familiar objects for a period of 10 min as the acquisition trial. Then, the animals were returned to the home cage for a 1-h inter-trail interval. In the test trial, each animal was returned to the same apparatus with a familiar object and a novel object for a 10 min period of exploration. The behavior of the animals was video recorded for subsequent analysis by EthoVision XT 13 software (Noldus). The NOR behavior was expressed as discrimination index, which was calculated as: the time spent on exploring the novel object / total exploration time (the total time spent on both familiar object and novel object).

### Translating Ribosome Affinity Purification (TRAP) followed by RNA sequencing (TRAP-Seq) using CCR2-CreER; R26R-EGFP/Rpl10A (CCR2-TRAP) mice

The 2 or 14 d post-LPS/HI mouse brains were harvested in ice-cold dissection buffer following previously described procedures to purify the cell-type specific translating RNAs 35. The rabbit anti-GFP antibodies, chromatin immunoprecipitation (ChIP) grade (#ab290, Abcam), were used for immunopurification. The purification specificity from CCR2-TRAP mice has been characterized 9. The cDNA library was generated with a TruSeq Stranded mRNA Library Prep Kit (Illumina) with Agencourt AMPure XP beads for PCR cleanup. Samples were loaded onto a NextSeq 500 mid-output 150 cycle cartridge and sequenced on a NextSeq 500 (Illumina).

### RNA scope assay

Mouse brain was freshly perfused and fixed in 4% PFA for 24 hours at 4 °C. After fixation, the brain was immersed in 30% sucrose. The tissue was frozen in the OCT embedding media with dry ice. The blocks were sectioned by cutting 20-μm sections. RNA probe is commercially available from Advanced Cell Diagnostics (ACD). Here, we used probes against mouse *Sall1* (ACD catalog no. 469661), *Ms4a7* (ACD catalog no #314601-C3, ACDBio), and positive control probe (ACD catalog no. 310771) and negative probe (ACD catalog no. 310043), and then performed the assay by using the RNAscope Fluorescent Multiplex Reagent Kit (ACD catalog no. 320850) according to the manufacturer's instructions.

### Statistical analysis

Statistical analysis was performed using one-way ANOVA followed by the Tukey test or unpaired t-test for two sample groups (GraphPad Prism). The mortality rate analysis in LPS/HI insult was performed using the Kaplan-Meier method. P values less than 0.05 were considered a significant difference. All values were expressed as mean ± SEM.

## Results

### Murine monocytes migrate across the choroid plexus into late embryonic brains

**Figure 1:**
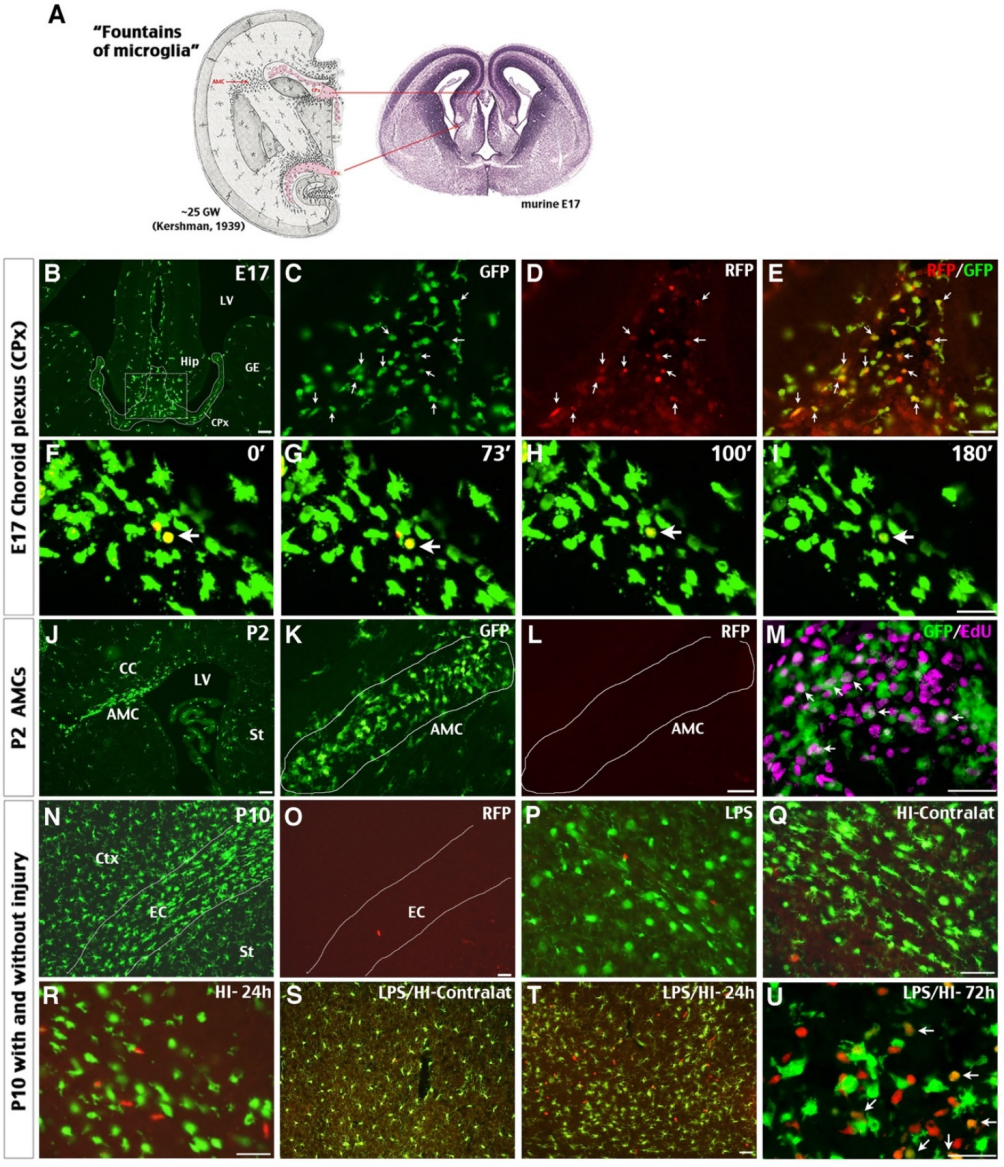
** Possible influx of CCR2^+^ monocytes in perinatal brains and neonatal injury. (A)** The classical view of “fountains of microglia” near the choroid plexus (CPx) and as amoeboid microglial cells (AMC) beneath the corpus callosum in the 25th gestational week (GW) of human fetus and their correlates in E17 mouse embryos (left is modified from Kershman, 1939 33). **(B-E)** Brains of E17 CCR2^RFP/+^; CX3CR1^GFP/+^ (R/G) embryos contained clusters of GFP^+^, RFP^+^, and RFP^+^/GFP^+^ double-positive cells in the CPx between the two primordial hippocampi (Hip). Shown are typical images in three embryos; arrows were added to compare the GFP- and/or RFP- expression. **(F-I)** Time-lapsed live imaging of E17 R/G mouse brain slice showed disappearance of RFP fluorescence in RFP^+^/GFP^+^ cells within 2 hours in the CPx (arrow). **(J-M)** The subcortical AMCs in P2 R/G mice were CX3CR1^GFP+^, but CCR2^RFP-^, and often co-labeled with anti-EdU (5-ethynyl-2'-deoxyuridine, arrows in M) that was injected one hour earlier. **(N-U)** The P10 R/G mouse brains contained numerous CX3CR1^GFP+^ microglia, but only scant CCR2^RFP+^ cells, when they were uninjured (N-O) or received an intraperitoneal injection of 0.3 mg/kg LPS 23 h earlier (P). **(Q-U)** Both pure-HI and LPS/HI injuries induced CCR2^RFP+^ cell infiltration in the ipsilateral cerebral cortex 24 h later (R-T), but not in the contralateral hemisphere (Q-S). Many amoeboid RFP^+^/GFP^+^ cells appeared in the ipsilateral cerebral cortex at 72 h after LPS/HI injury (arrows in U). CC: corpus callosum; Ctx: cortex; EC: external capsule; GE: ganglionic eminence; LV: lateral ventricle; St: striatum. N = 3 for each time point. Scale bar: 50 μm.

We first examined the presence of CCR2^RFP+^ monocytes in the choroid plexus (CPx) lining the third ventricle in E17.5 CCR2^RFP/+^; CX3CR1^GFP/+^ (hereafter referred to as R/G) embryos, since this is a major “microglia fountain” site in classical literature 33 (Figure 1A). Indeed, CCR2^RFP+^ and CCR2^RFP+^/CX3CR1^GRP+^ double-positive cells intermingled with amoeboid CX3CR1^GFP+^ cells were detected in the CPx between the two primordial hippocampi (Hip) (Figure 1B-E; n = 3). Live-imaging of E17.5 R/G brain slices showed that the RFP expression in CCR2^RFP+^ CX3CR1^GRP+^ cells disappeared within two hours (Figure 1F-I). In contrast, no CCR2^RFP+^ cells were detected among the subcortical amoeboid microglial cells (AMCs) - another classic “microglial fountain” site 33 —that were filled with CX3CR1^GFP+^ and EdU^+^ amoeboid cells at postnatal day 2 (P2) in R/G mice (Figure 1J-M). These results suggest that CCR2^+^ monocytes enter late embryonic mouse brains through selective locations such as the CPx, and rapidly become CCR2^-^ CX3CR1^+^ amoeboid cells before wide dispersion inside the neuroepithelium. These data also support our report that CCR2^+^ monocytes may differentiate into P2RY12^+^ microglia in the perinatal period 9.

By P10, the parenchyma of cerebral cortex (Ctx) and striatum (St) and the external capsule (EC) in R/G mice were populated by bipolar or ramified CX3CR1^GFP+^ microglia, but without CCR2^RFP+^ cells (Figure 1N-O; n = 3). Intraperitoneal injection of low-dose LPS (0.3 mg/kg) did not increase CCR2^RFP+^ cells in brain parenchyma of P10 R/G mice (Figure 1P; n = 3). In contrast, abundant CCR2^RFP+^ cells were detected in the ipsilateral cerebral cortex at 24 h after the unilateral HI and LPS-sensitized HI insult (Figure 1Q-T; n = 3 for each group). Further, many CCR2^RFP+^/CX3CR1^GRP+^ double-positive cells appeared at 72 h after LPS/HI injury (arrows in Figure 1U; n = 3). These data suggest that both HI and LPS/HI injury attract blood-borne monocytes into the neonatal brains.

### LPS/HI injury accumulates CCR2^RFP+^ CX3CR1^GFP+^ monocytic cells expressing inflammatory cytokines in neonatal brains

**Figure 2:**
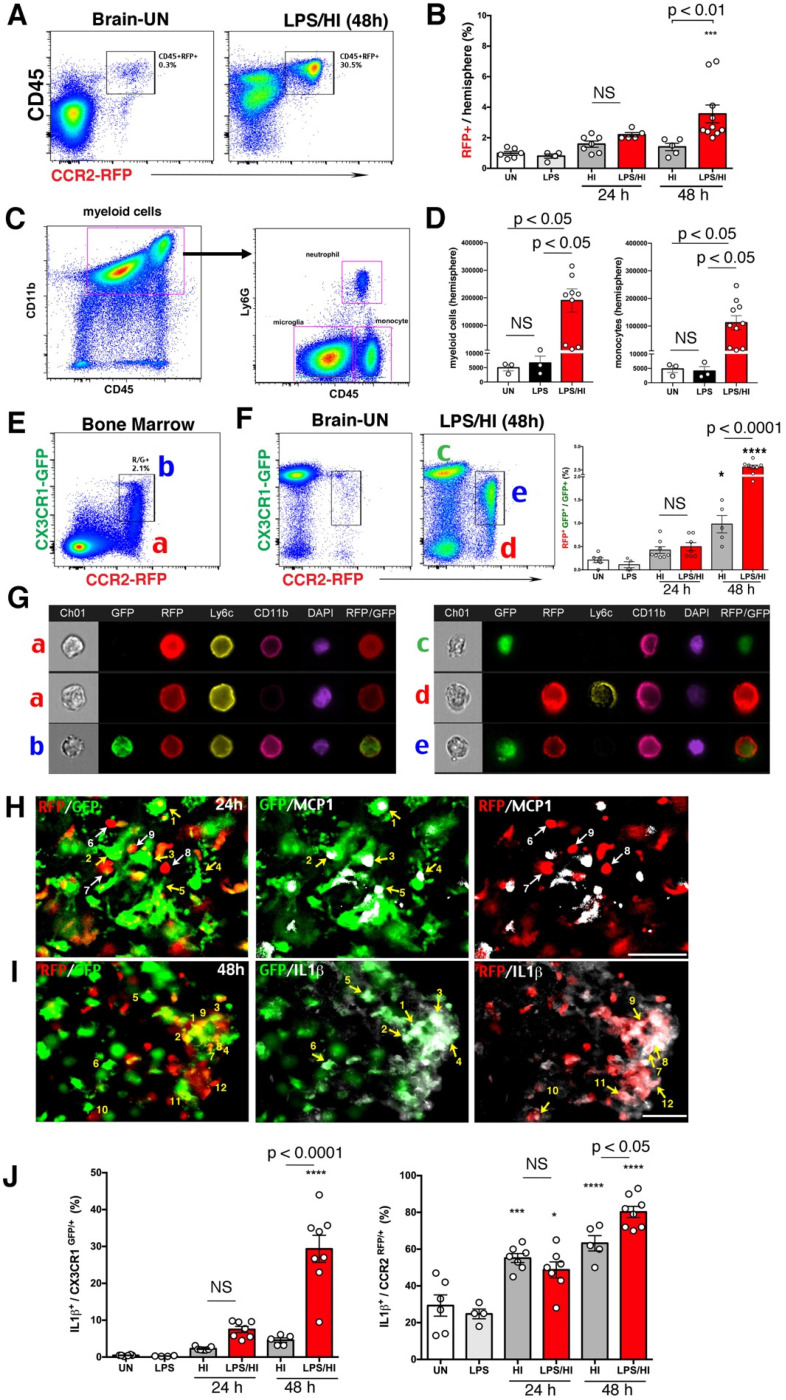
** CCR2^RFP+^ CX3CR1^GFP+^ monocytic infiltrates in neonatal R/G mouse brains. (A-B)** Flow cytometry analysis showed more CD45^hi^ CCR2^RFP+^ cells in LPS/HI- than HI-injured ipsilateral hemisphere at 48 h (p < 0.01), but not at 24 h post-injury in P10 R/G mice. Low-dose LPS injection failed to induce CD45^hi^ CCR2^RFP+^ cells compared with uninjured (UN) R/G mice. **(C-D)** P10 wildtype mouse brains also showed a significant increase of CD45^hi^ CD11b^+^ myeloid cells and CD45^hi^ CD11b^+^ Ly6G^-^ monocytes (p < 0.05 for both), when compared to uninjured or LPS-injected mice. E-G Dot-plot flow cytometry (**E-F**) and typical imaging-flow patterns (**G**) of the bone marrow and brain in P10 R/G mice. The RFP^+^ cells in bone marrow were CCR2^RFP+^ CX3CR1^GFP-^Ly6C^+^, but CD11b^+^ or CD11b^-^(indicated as a). The RFP^+^/GFP^+^ double-positive cells in bone marrow were CCR2^RFP+^ CX3CR1^GFP-^ Ly6C^+^ CD11b^+^ (indicated as b). The GFP^+^ cells in the brain at 48 h post-LPS/HI were CCR2^RFP-^ CX3CR1^GFP+^ Ly6C^-^ CD11b^+^, typical for microglia (indicated as c). The RFP^+^ cells in the brain were CCR2^RFP+^ CX3CR1^GFP-^ Ly6C^+^ CD11b^+^, as expected for pro-inflammatory monocytes (CX3CR^lo^ Ly6C^hi^) (indicated as d). The more numerous RFP^+^/GFP^+^ cells in the brain were CCR2^RFP+^ CX3CR1^GFP+^ Ly6C^-^ CD11b^+^, consistent with patrolling monocytes (CX3CR1^hi^ Ly6C^lo^) or pro-inflammatory monocytes with diminished Ly6C-expression (indicated as e). Note the increase (p < 0.0001) of RFP^+^/GFP^+^ cells among GFP^+^ in LPS/HI- over HI-injured brains at 24 h recovery. **(H)** GFP^+^-only microglia (numbered 1-5), but not RFP^+^-only monocytes (numbered 6-9), expressed MCP1 (white) at 24 h post-LPS/HI injury. **(I)** Both GFP^+^- (numbered 1-6) and RFP^+^- (numbered 7-12) amoeboid cells expressed IL-1β (white) in the 48 h post-LPS/HI brain. **(J)** Quantification by flow cytometry indicated that the dual-LPS/HI insult lead to more IL-1β-expression than pure-HI insult in CX3CR1^GFP+^ (p < 0.0001) and CCR2^RFP+^ cells (p < 0.05) at 48 h post-injury. Data are shown as mean ± SEM. Statistic comparisons were performed using one-way ANOVA, followed by B-K-Y adaptive FDR control (D) or Tukey (B, F and J) post-hoc analysis for multiple comparisons. NS: not significant, * p < 0.05, *** p < 0.001 and **** p < 0.0001 versus UN. N = 4 mice for each time point. Scale bar: 50 μm.

Flow cytometry showed that LPS/HI injury attracted more CD45^hi^ CCR2^RFP+^ monocytes in ipsilateral hemisphere at 48 h, but not at 24 h, than pure-HI injury (Figure 2A-B). Confirmation experiment in wildtype mice showed an increase to ~190,000 CD45^hi^ CD11b^+^ myeloid cells, including ~110,000 CD45^hi^ CD11b^+^ Ly6G^-^ monocytes, in the ipsilateral hemisphere at 48 h post-LPS/HI (Figure. 2C-D). Next, we used R/G neonates for imaging-flow analysis, which revealed two subtypes of CCR2^RFP+^ cells in the bone marrow, namely R (singular CCR2^RFP+^) and R/G cells (CCR2^RFP+^/CX3CR1^GFP+^ double-positive, Figure 2E), as well as, three groups, G (singular CX3CR1^GFP+^), R, and R/G cells, in brain parenchyma (Figure 2F). Notably, the percentage of RG cells among GFP^+^ cells was significantly higher in the ipsilateral brain parenchyma at 48 h after LPS/HI than pure-HI injury (Figure 2F). Imaging-flow analysis showed that both R and R/G cells in bone marrow express Ly6C, as typical for the monocyte lineage, and G cells in the post-LPS/HI brain were CCR2^RFP-^ CX3CR1^GFP+^ Ly6C^-^ CD11b^+^, as expected for microglia. In contrast, the R cells in post-LPS/HI brain were CCR2^RFP+^ CX3CR1^GFP-^ Ly6C^+^ CD11b^+^, consistent with infiltrating CX3CR1^-^ Ly6C^hi^ monocytes, but the R/G cells in brain were CCR2^RFP+^ CX3CR1^+^ Ly6C^-^ CD11b^+^, suggesting that they were either patrolling Ly6C^lo^ monocytes or a subset of Ly6^hi^ monocytes in transition to CX3CR1^+^ Ly6C^-^ microglia-like cells through *in-situ* programming 22 (Figure 2G).

Next, we used fluorescence staining to compare the expression of pro-inflammatory cytokines by CX3CR1^GFP+^ and CCR2^RFP+^ cells in the R/G mouse brain at 24 h post-LPS/HI. This analysis revealed the expression of monocyte chemoattractant protein-1 (MCP1) by CX3CR1^GFP+^ microglia (cells labeled as 1-5 in Figure 2H), but not CCR2^RFP+^ monocytes (labeled as 6-9 in Figure 2H). In contrast, both CX3CR1^GFP+^ cells (labeled as 1-6 in Figure 2I) and CCR2^RFP+^ monocytes (labeled as 7-12 in Figure 2I) expressed IL-1β. Quantification showed that CX3CR1^GFP+^ microglia and a higher ratio of CCR2^RFP+^ monocytes expressed IL-1β at 48 h post-LPS/HI (Figure 2J). These results suggest that monocytic infiltrates express pro-inflammatory cytokines after LPS/HI brain injury, but their ability to express monocyte-recruiting cytokines may be more limited.

### Monocytic infiltrates assume a microglia-like morphology and expanded longevity in post-LPS/HI brains

**Figure 3:**
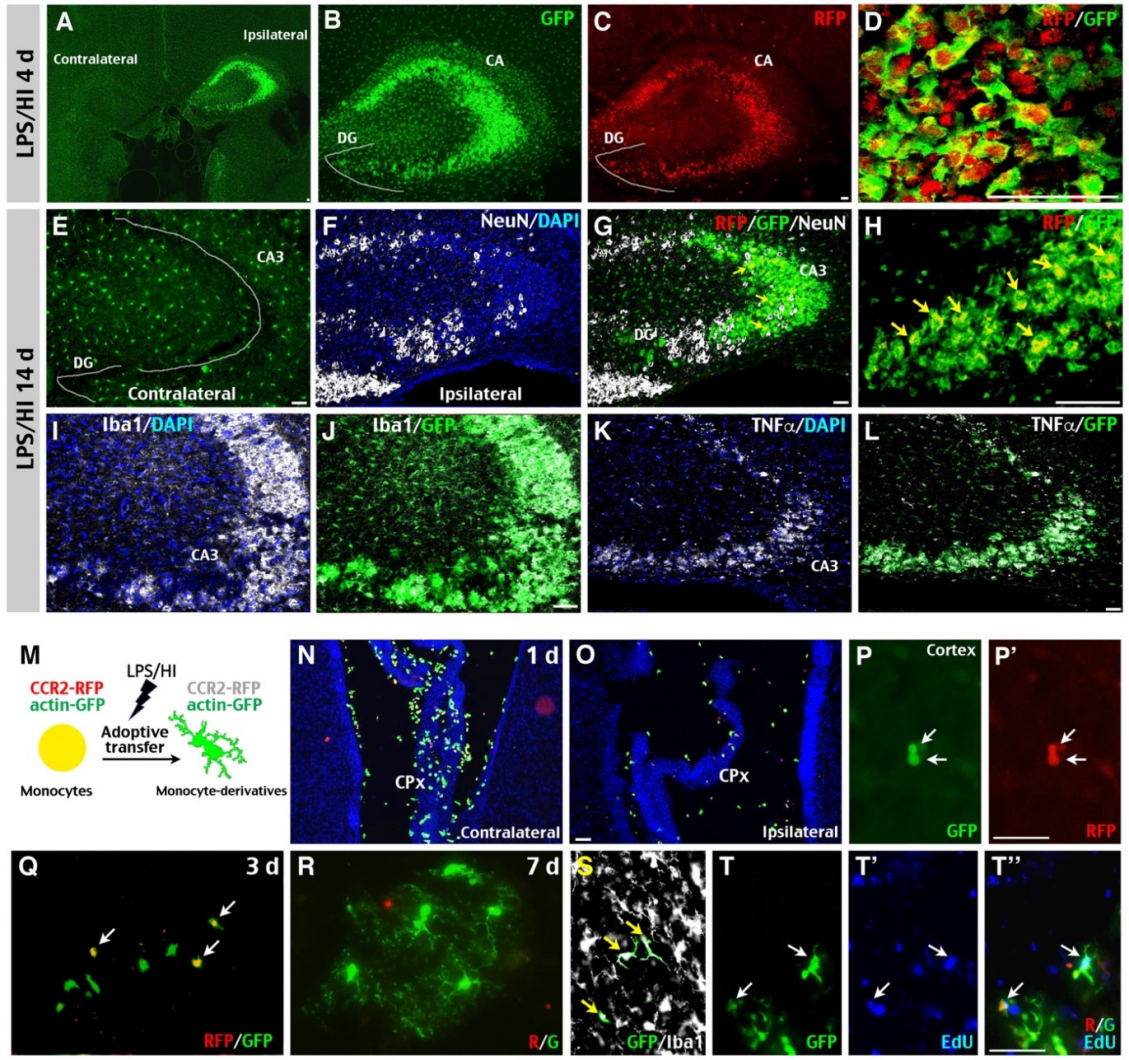
** Potential “monocyte-to-microglia” transition after neonatal LPS/HI injury. (A-D)** At 4 d post-LPS/HI, the ipsilateral hippocampus (mainly in the CA, but not DG subfield) was populated by GFP^+^, RFP^+^, and RFP^+^/GFP^+^ double-positive amoeboid cells, without obvious tissue damage in R/G mice. **(E-L)** GFP^+^, RFP^+^, and RFP^+^/ GFP^+^ amoeboid cells were still detectable in the ipsilateral CA3 subfield of few NeuN^+^ neurons in R/G mice at 14 d post-LPS/HI (F-H). In contrast, the contralateral hippocampus only showed ramified GFP^+^ microglia at 14 after LPS/HI (E). The majority of GFP^+^ amoeboid cells in ipsilateral CA3 subfield were Iba1^+^ (I-J) and TNFα^+^ (K-L) at 14 d post-LPS/HI. **(M-T'')** Intravenous grafting of CCR2^RFP+^; actin-GFP^+^ monocytes to wildtype P10 mice at 1 h after LPS/HI injury to assess their potential metamorphosis (M). At 1 d post-transfer, many round RFP^+^/ GFP^+^ double-positive cells were detected in the contralateral CPx (N) and, to a lesser degree, in ipsilateral CPx (O) and brain parenchyma (P, P'). At 3 d post-transfer, there were more GFP^+^-only than RFP^+^/ GFP^+^ double-positive amoeboid cells in the ipsilateral brain parenchyma (Q). At 7 d post-transfer, GFP^+^-only cells with a ramified microglia-like morphology were detected in the ipsilateral hemisphere (R). These GFP^+^-only microglia-like cells were Iba1^+^ (S) and often contained the EdU that was injected after adoptive transfer (T-T''). DG: dentate gyrus; CA: cornu ammonis. N = 3 mice for each time point in A-L, and n = 6 in N-P, each time point. Scale bar: 50 μm.

In our analysis of R/G mice, we noticed that the hippocampus in ipsilateral hemisphere often harbored many CCR2^RFP+^ monocytes post-LPS/HI, even without severe local destruction. As shown in Figure 3A-C, the ipsilateral hippocampus was heavily populated by CX3CR1^GFP+^ and CCR2^RFP+^ AMCs in the CA sector, but less in the DG subfield at 4 d post-LPS/HI (n = 3). Confocal microscopy showed that many CX3CR1^GFP+^ AMCs co-expressed the CCR2^RFP+^ marker (Figure 3D; n = 3). By 14 d post-LPS/HI, the ipsilateral hippocampus contained many CX3CR1^GFP+^ AMCs, few CCR2^RFP^/CX3CR1^GFP^ double-positive cells, and gaps of NeuN^+^ neurons in the CA3 subfield (Figure 3E-H; n = 3). Double-labeling showed that CX3CR1^GFP+^ AMCs in the CA3 subfield expressed Iba1 (Figure 3I-J) and TNFα (Figure 3K-L; n = 3) at 14 d post-LPS/HI. Since the lifespan of monocytes is ~2 days in the blood 12, the presence of CCR2^RFP^/CX3CR1^GFP^ double-positive cells in the hippocampus 2 weeks after LPS/HI raised the possibility that some emigrant monocytes may attain a longer lifespan and microglia-like properties inside the injured brain.

To test this possibility concerning the outcomes of monocytic infiltrates in LPS/HI-injured brains, we purified monocytes from the bone marrow of CCR2^RFP/+^; actin-GFP mice and injected them intravenously into wildtype mouse neonates post-LPS/HI (Figure 3M). At 1 d after transfer, many round RFP^+^/GFP^+^ double-positive cells—as expected for CCR2^RFP/+^; actin-GFP monocytes—were detected in the CPx, but not in the parenchyma of contralateral hemisphere (Figure 3N; n = 6). The ipsilateral hemisphere showed a reverse pattern with more RFP^+^/GFP^+^ cells in parenchyma than in the CPx (Figure 3O-P'; n = 6). At 3 d post-transfer, more GFP^+^ than RFP^+^/GFP^+^ AMCs were found in ipsilateral hemisphere (Figure 3Q; n = 6). By 7-day post-transfer, many ramified, microglia-like GFP^+^ Iba1^+^ cells were detected in the ipsilateral hemisphere (Figure 3R-S; n = 6). Further, many of these GFP^+^ cells contained the DNA-analog EdU injected over 2 days post-transfer (Figure 3T-T''). Together, these results suggest that some CCR2^+^ monocytes gradually adopt a microglia-like morphology and lost CCR2 expression in the LPS/HI-injured brain parenchyma.

### CCR2-CreER-based fate-mapping suggests long-term survival of monocytic infiltrates

**Figure 4:**
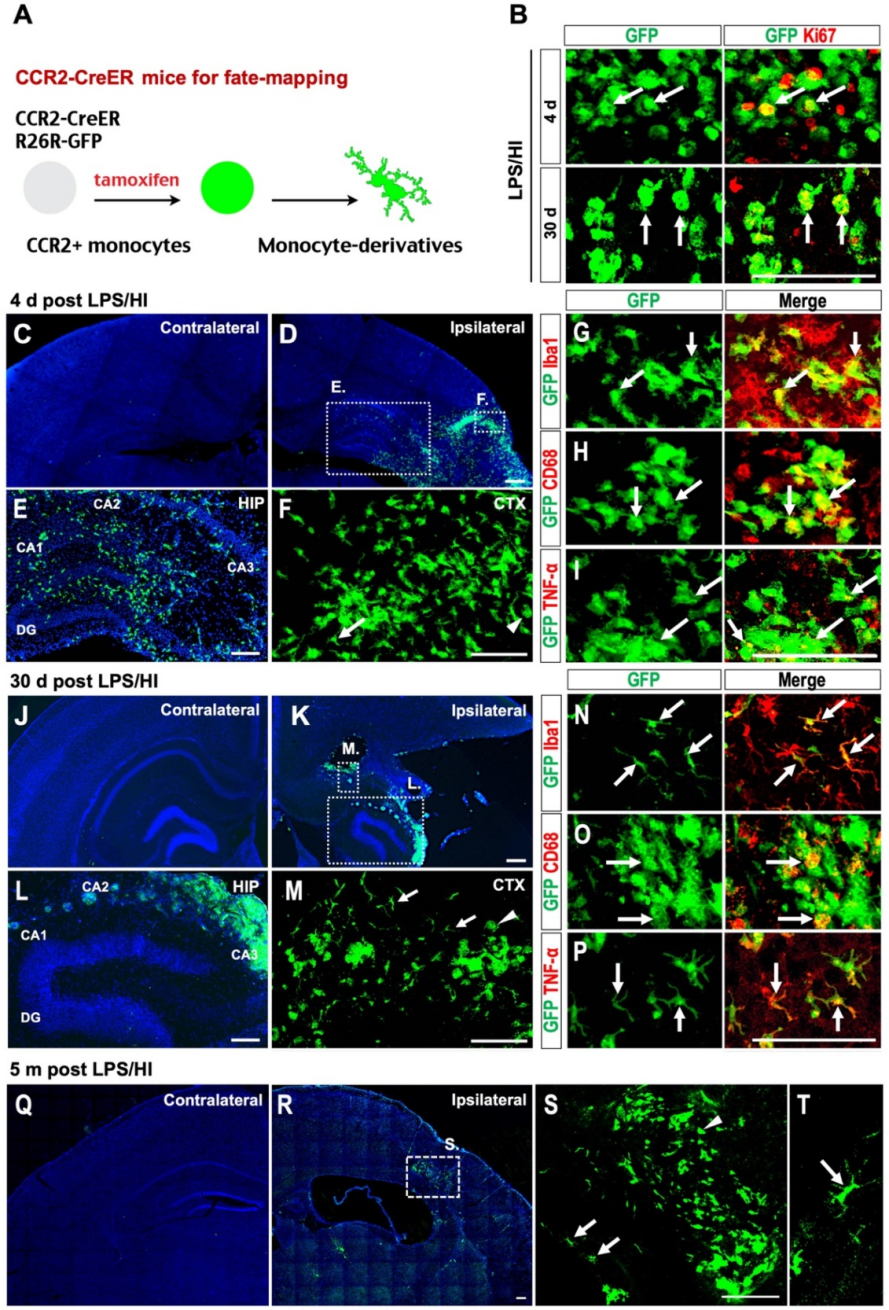
** Fate-mapping monocytes in LPS/HI-injured CCR2-CreER; R26R-GFP mice. (A)** Scheme of monocytic fate-mapping via CCR2-CreER; R26R-GFP mice. Tamoxifen-dosing labels CCR2^+^ monocytes and their derivatives with constitutive GFP fluorescence regardless of the CCR2 promoter activity. **(B)** GFP^+^ monocytic infiltrates exhibited stronger anti-Ki67 immuno-signals at 4 d than 30 d post-LPS/HI. **(C-F)** GFP^+^ monocytic infiltrates were found in ipsilateral hemisphere at 4 d post-LPS/HI (D), including the hippocampus (E) and cerebral cortex (F), but not in contralateral hemisphere (C). Magnified images of the ipsilateral cortex and hippocampus showed both amoeboid (arrowhead) and ramified morphology (arrow) of GFP^+^ cells. (E, F) are magnified images of the squares shown in (D). **(G-I)** Immunostaining showed that many GFP^+^ monocytic infiltrates expressed Iba1 (G), CD68 (H) and TNFα (I) at 4 d post-LPS/HI. **(J-P)** At 30 d post-LPS/HI, GFP^+^ monocytic infiltrates were still absent in contralateral hemisphere (J), but found in the damaged ipsilateral hemisphere (K), including the hippocampus (L) and cerebral cortex (M). More GFP^+^ monocytic derivatives displayed a ramified microglia-like morphology (arrows in M). (L, M) are magnified images of squares shown in (K). **(N-P)** Immunostaining shows that ramified GFP^+^ monocytic derivatives expressed Iba1 (N) and TNFα (P), but only amoeboid GFP^+^ derivatives expressed CD68 (O) at 30 d post-LPS/HI. **(Q-T)** At 5 months post-LPS/HI, GFP^+^ monocytic derivatives were still restricted in the ipsilateral hemisphere (Q-R), exhibiting both ramified (arrows in S, T) and fewer amoeboid morphology (arrowhead in S). (S) is magnified image of square shown in (R). N = 3 mice for each time point in A-B, n = 6 in C-F, and n = 3 in Q-T. Scale bar: 50 μm (B-P), 100 μm (Q-T).

Next, we used CCR2-CreER; R26R-EGFP (Ai6) mice to track emigrant monocytes after neonatal LPS/HI brain injury 9. This method labels CCR2^+^ monocytes and their derivatives by constitutive GFP expression in a tamoxifen-induced manner, while no GFP^+^ cells exist in the CCR2-CreER; R26R-GFP mouse brains without tamoxifen treatment (Figure 4A). When tamoxifen was applied to CCR2-CreER; R26R-GFP mice for 2 days prior to LPS/HI, around 20% GFP^+^ cells expressed Ki67 in the ipsilateral cortex at 4 d post-LPS/HI, which dropped to <10% at 30 d post-LPS/HI, suggesting that the infiltrated monocytes may gradually exit an active cell-cycle in the brain (Figure 4B and Figure S1A; n = 3).

At 4 d post-LPS/HI, GFP^+^ cells were detected in the ipsilateral cerebral cortex (CTX) and hippocampus (HIP) (Figure 4C-F; n = 6), but not in contralateral hemisphere. These GFP^+^ cells predominantly displayed an amoeboid cell-shape and expressed Iba1 (Figure 4G). Many also expressed pro-inflammatory markers, such as CD68 and TNFα (arrows in Figure 4H-I; n = 6). By 30 d post-LPS/HI, GFP^+^ cells remained confined in the ipsilateral hemisphere, but showed two distinct configurations: either conglomerates of AMCs or individual microglia-like cells with a ramified morphology (Figure 4J-M; n = 6). While only AMC-like GFP^+^ cells were CD68^+^ (a macrophage marker) at this stage, many ramified GFP^+^ Iba1^+^ cells expressed TNFα^+^, suggesting pro-inflammatory actions 36 (Figure 4N-P; n = 6). Finally, at 5 months after LPS/HI, the ipsilateral but not contralateral cortex still harbored many GFP^+^ AMCs and GFP^+^ microglia-like cells (Figure 4Q-T; n = 3). These fate-mapping results also suggest that some emigrant monocytes may contribute to the resident microglia pool after LPS/HI injury.

### Monocytic infiltrates have long-lasting deleterious effects in LPS/HI-injured brains

**Figure 5:**
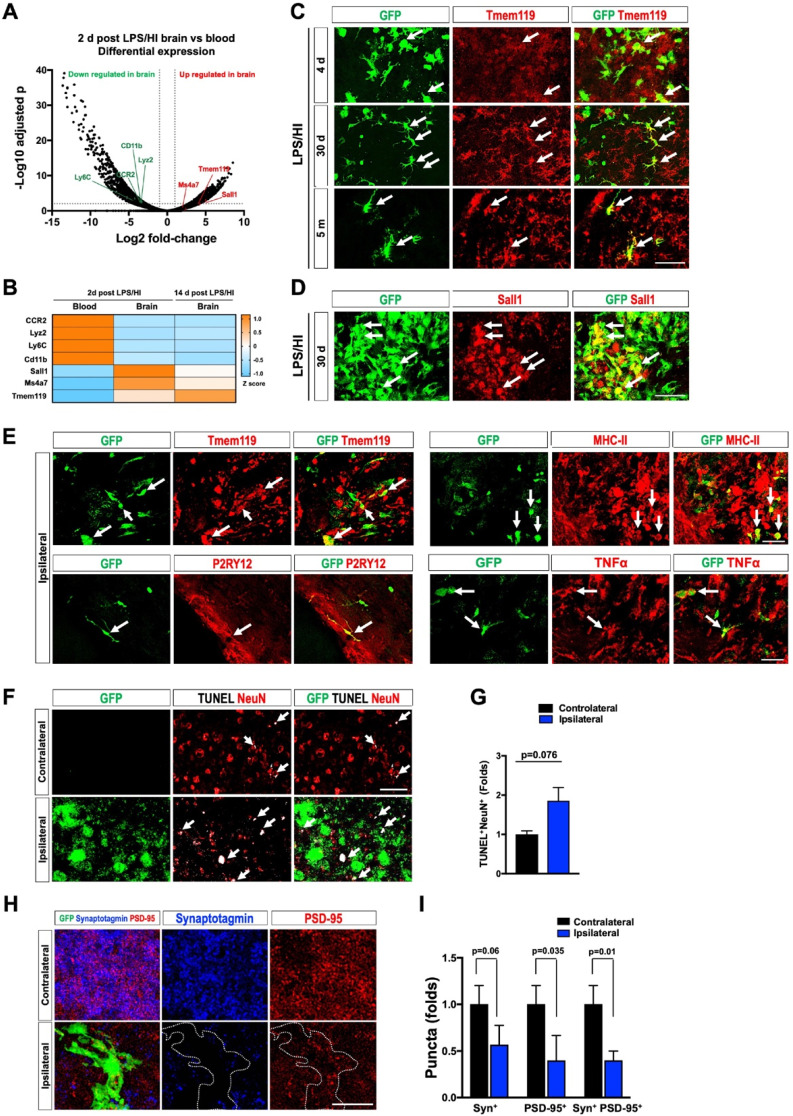
** Protracted harmful effects of monocytic infiltrates after LPS/HI brain injury. (A)** Volcano plot of differentially expressed genes (DEGs) between monocytic derivatives in the 2 d post LPS/HI blood (left) and brain (right), based on TRAP-based RNA-sequencing in tamoxifen-doses CCR2-CreER; R26R-EGFP/Rpl10A mouse neonates. The vertical dash-lines indicate ± 2-fold change, and the horizonal dash-line denotes p < 0.05. Selected marker genes for monocytes (green) and microglia (red) were labeled. **(B)** The z-score display of selected monocyte or microglia marker gene expression in the blood or post-LPS/HI brain. Note the expression of monocytic genes (CCR2, Lyz2, Ly6C, and CD11b) were reduced, while the microglia marker genes (Sall1, Ms4a7, and Tmem119) were up-regulated in 2 d and 14 d post-LPS/HI brain. **(C-E)** Double-labeling showed more specific expression of Tmem119 (C) and *Sall1* mRNA (D) in 30 d post-LPS/HI CCR2-CreER; R26R-GFP mouse brain. At 5 m post-LPS/HI (E), amoeboid GFP^+^ derivatives expressed MHC-II, TNFα, and microglial markers (Tmem119 and P2RY12). **(F-G)** At 30 d post-LPS/HI, the ipsilateral hippocampus contained more TUNEL^+^/NeuN^+^ double-positive nuclei, mostly surrounded by GFP^+^ monocytic infiltrates (arrows), than the contralateral counterpart (p = 0.02 by unpaired t-test). **(H-I)** Similarly, the ipsilateral hippocampus showed significant reduction of anti-PSD-95^+^ (p = 0.035) and colocalized anti-synaptotagmin (Syn)^+^/anti-PSD-95^+^ puncta (p = 0.01), but not anti-Syn^+^ puncta (p = 0.06), than contralateral hippocampus at 30 d after LPS/HI injury. Note the area harboring GFP^+^ monocytic derivatives (marked by dash-line) showed the greatest reduction of anti-Syn and anti-PSD-95 signals. N = 4 mice per group in A-B. N = 3 for each time point in C-I. Results are displayed as the mean ± SEM. Statistics are performed as unpaired *t*-test (G and I). Scale bar: 50 μm.

We also produced CCR2-CreER; R26R-EGFP/Rpl10a (hereafter referred to as CCR2-TRAP) mice to determine the transcriptome of emigrant monocytes using the translating ribosome affinity purification (TRAP) protocol for RNA-Sequencing 35. We first compared the transcriptome of monocytes in the blood versus in the brain at 2 d post-LPS/HI in tamoxifen-dosed CCR2-TRAP mice (Figure 5A; 4 mice in each group were pooled). This analysis showed that *CD11b*, *Lyz2*, *CCR2*, and *Ly6C* transcripts (markers for circulating monocytes) declined, whereas *Tmem119*, *Ms4a7*, and *Sall1* mRNAs (all are microglial marker genes 37-39) were increased in the brain-infiltrated monocytes (Figure 5A). When the third group of monocytes at 14 d post-LPS/HI in tamoxifen-dosed CCR2-TRAP mice was included for comparison, they showed the highest level of *Tmem119* transcripts (a microglial signature marker 38) (Figure 5B).

Immuno-labeling also suggested an ascending expression of Tmem119 by monocyte derivatives from 4 d to 30 d to 5 months in the CCR2-CreER; R26R-GFP mouse brains after LPS/HI injury (Figure 5C, n = 3). *In-situ* hybridization confirmed the expression of *Sall1* and *Ms4a7* by monocytic derivatives in the ipsilateral cortex at 30 d after LPS/HI (Figure 5D and Figure S1B; n = 3). CCR2^+^ monocyte-derivatives also expressed the microglial markers (Tmem119 and P2RY12) or pro-inflammatory markers (MHC-II and TNFα) at 5 months after LPS/HI (Figure 5E and Figure S1C; n = 3). Together, these results suggest long-lasting harmful effects by some monocytic infiltrates in LPS/HI-injured brains.

Consistent with this notion, the ipsilateral hippocampal CA3 subfield contained more NeuN^+^/TUNEL^+^ double-positive nuclei than contralateral counterpart at 30 d post-LPS/HI (Figure 5F-G; n = 3). Confocal microscopy analysis of anti-synaptotagmin (Syn) and anti-PSD-95 labeling showed a significant reduction of PSD-95^+^ and Syn^+^/PSD-95^+^ puncta in the ipsilateral CA3 subfield at 30 d post-LPS/HI, particularly within the area populated by GFP^+^ monocyte derivatives (white outlines in Figure 5H-I; n = 3). These results suggest long-term deleterious effects by monocytic infiltrates (e.g. enhanced synaptic pruning and neuronal cell death) after neonatal LPS/HI brain injury 40.

### Deletion of the CCR2 receptor reduces monocytic influx and LPS/HI brain damage

**Figure 6:**
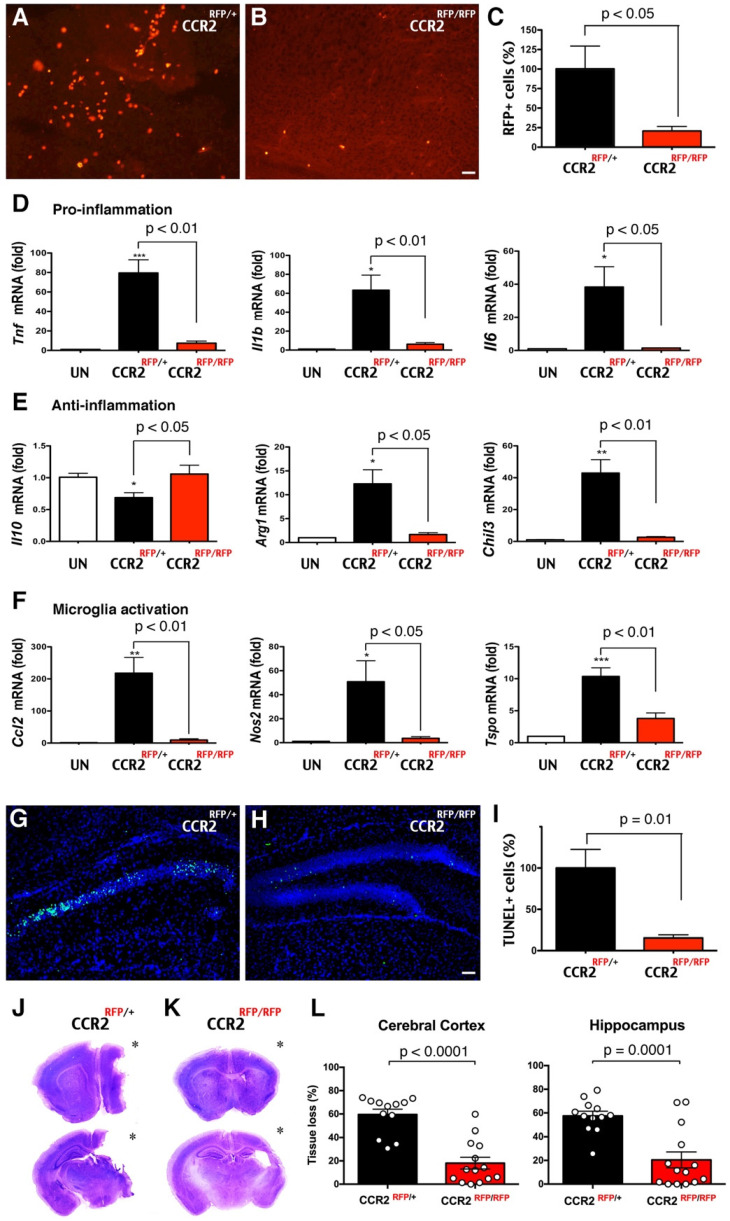
** Reduction of LPS/HI brain damage in mice lacking CCR2 in monocytes. (A-C)** Homozygous CCR2^RFP^/^RFP^ mice contained significantly fewer RFP^+^ monocytes in 24 h post-LPS/HI brain than CCR2^RFP^/^+^ mice (p < 0.05 by unpaired *t*-test, n = 6 for each). **(D-F)** LPS/HI injury significantly the brain *IL-10* mRNAs, but elevated those for pro-inflammation (*Tnf, Il1b* and* Il6*), anti-inflammation (*Arg1* and* Chil3*) and microglia activation (*Ccl2*, *Nos2* and *Tspo*) mRNAs in CCR2^RFP/+^ mice. These post-LPS/HI gene expression alterations were attenuated in CCR2^RFP/RFP^ mice. * p < 0.05, ** p < 0.01, *** p < 0.001 versus UN. N >4 for each group. **(G-I)** CCR2^RFP/RFP^ mice showed ~90% reduction of TUNEL^+^ cell death in the ipsilateral hippocampus than CCR2^RFP/+^ mice at 1 d post-LPS/HI (p = 0.01 by unpaired t-test, n = 4 for each). **(J-K)** Representative images of CCR2^RFP/+^ and CCR2^RFP/RFP^ mouse brains at 7 d post-LPS/HI injury. **(L)** CCR2^RFP/RFP^ showed significantly less tissue loss than CCR2^RFP/+^ mice in the cerebral cortex (59.5 ± 4.6% vs 18.1 ± 5.0%) and hippocampus (57.4 ± 4.0% vs 20.4 ± 6.8%) at 7 d post-LPS/HI injury. N = 12 for CCR2^RFP/+^ and n = 14 for CCR2^RFP/RFP^ mice. Asterisk indicates the ipsilateral hemisphere. Results are displayed as the mean ± SEM. Statistics are performed as unpaired *t*-test (C, I and L) and one-way ANOVA following by Tukey post-hoc analysis (D, E and F). Scale bar: 50 μm.

Next, we assessed the functions of CCR2^+^ monocytes in LPS/HI brain injury. Since CCR2, the receptor for monocyte chemoattractant protein-1 (MCP-1), often guides the infiltration of monocytes to injured tissue, we compared the responses to LPS/HI injury between CCR2^RFP/+^ and CCR2^RFP/RFP^ mouse neonates. In CCR2^RFP/RFP^ knock-in mice, monocytes are present but cannot respond to MCP-1 13. We found that the numbers of RFP^+^ cells were significantly diminished from CCR2^RFP/+^ to CCR2^RFP/RFP^ mouse brain at 24 h post-LPS/HI (Figure 6A-C; n = 6 for each). The reduction of migrant CCR2^RFP+^ monocytes correlated with fewer mRNAs of pro- and anti-inflammatory cytokines (*Tnf, Il1b, Il6, Arg1, Chil3*, but not *Il10*) and microglial activation markers (*Ccl2, Nos2,* and *Tspo*) in ipsilateral hemisphere at 24 h 41 (Figure 6D-F; n = 4-6 for each group). The homozygous CCR2^RFP/RFP^ neonates also exhibited 85% reduction of TUNEL^+^ cell death in ipsilateral hippocampus at 24 h post-LPS/HI (Figure 6G-I; n = 4 for each). By 7 d post-LPS/HI, CCR2^RFP/RFP^ mice showed a significant reduction of tissue loss in the ipsilateral cortex and hippocampus (Figure 6J-L; n = 12 for CCR2^RFP/+^ and n = 14 for CCR2^RFP/RFP^ mice). These results suggest that emigrant CCR2^+^ monocytes promote acute inflammation and brain damage after neonatal LPS/HI injury.

### Pharmacological inhibition of CCR2 opposes LPS/HI injury and cognitive impairment

**Figure 7:**
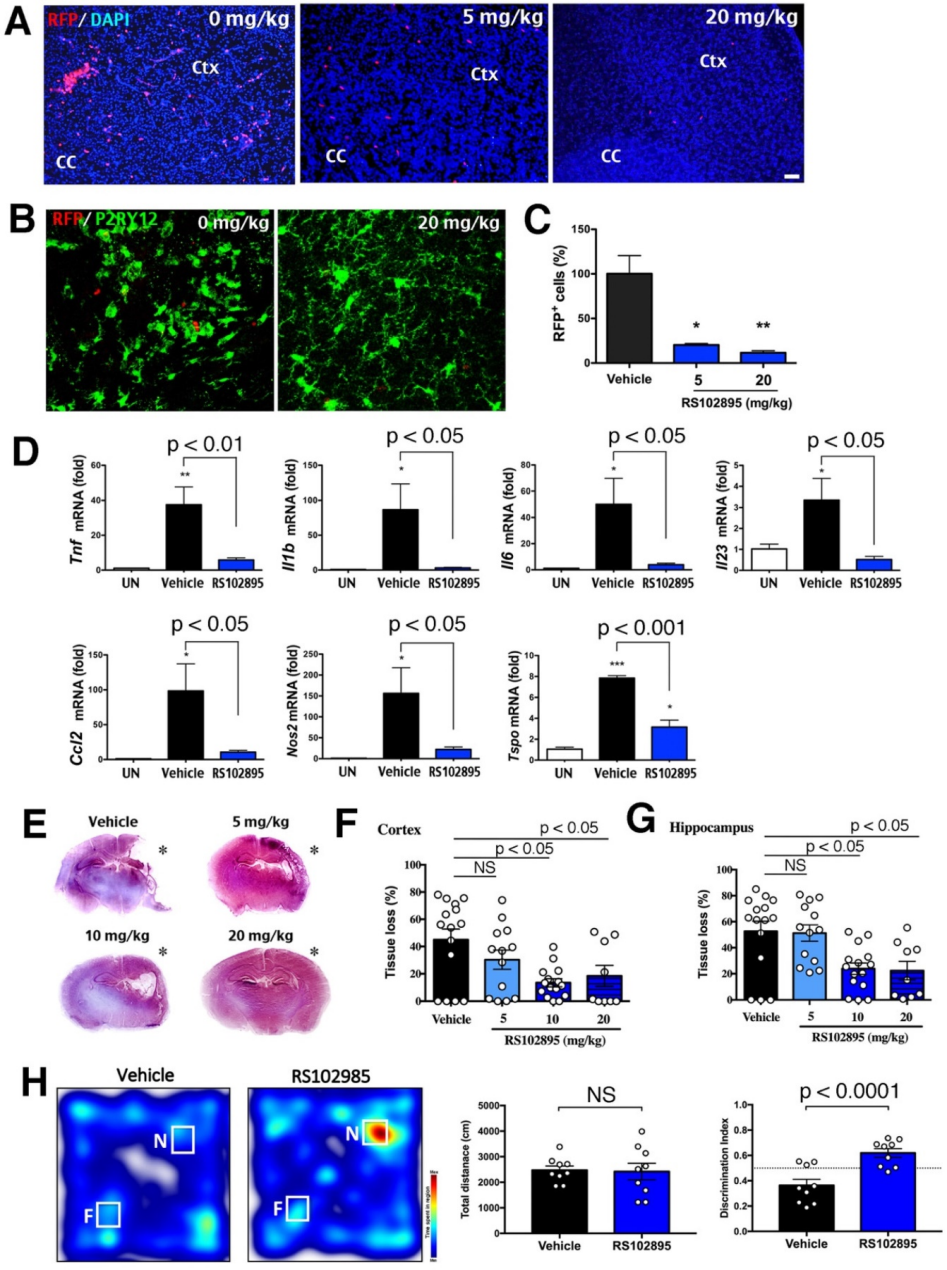
** Protection against neonatal LPS/HI injury with CCR2-inhibitor treatment. (A, C)** CCR2^RFP/+^ mice receiving two doses of 5 or 20 mg/kg RS102895 (IP) within 1 h post-LPS/HI contained significantly fewer RFP^+^ cells than vehicle-treated mice 24 h later (n = 8 for vehicle, n = 3 for RS102895-5 mg/kg and n = 5 for RS102895-20 mg/kg). (B) RS102895 (20 mg/kg x 2) treatment showed more ramified microglia by Tmem119 staining than the vehicle group. Scale bar: 50 μm. **(D)** RT-qPCR analysis shows that RS102895 treatment (10 mg/kg) significantly attenuated LPS/HI-induced *Tnf, Il1b*, *Il6*, *Il23*, *Ccl2*, *Nos2* and *Tspo* transcripts at 24 h recovery (n = 4 for untouched, n = 4 for vehicle and n = 3 for RS102895-10mg). **(E)** Representative images of vehicle-versus-RS102895 treated C57BL/6 mice at 7 d after neonatal LPS/HI injury. **(F-G)** Quantification shows significant reduction of cerebral cortex (F) and hippocampus (G) tissue loss in the mice receiving 5, 10 or 20 mg/kg RS102895 treatment (2 doses), when compared to vehicle-treated mice (n = 15 for vehicle, n = 13 for RS102895-5mg, n = 15 for RS102895-10mg and n = 9 for RS102895-20mg). **(H)** Representative heat map of the time spent with a novel (N) or familiar (F) object by vehicle-versus-RS102896 treated mice (10 mg/kg) at 45 d post-LPS/HI injury. Quantification showed an improved discrimination index (the ratio of time spent with the novel over the time with the familiar object) in RS102896-treated mice compared with vehicle-treated mice (p < 0.0001 by unpaired t-test; n = 9 for each group). The dash-line indicates a discrimination index at 0.5. * p < 0.05, ** p < 0.01 and *** p < 0.001 versus vehicle-treated control by one-way ANOVA with Tukey post-hoc analysis.

Given the genetic proof-of-principle evidence, we sought to determine whether the application of CCR2-inhibitor (RS102895 42) also reduces neonatal LPS/HI brain damage. Two doses of 5 or 20 mg/kg RS102895 by IP-injection within 1 h post-LPS/HI significantly decreased the number RFP^+^ monocytes at 24 h in CCR2^RFP/+^ neonates (Figure 7A, C; n>3 each group). The RS102895 treatment also diminished microglial activation, resulting in a reduction of amoeboid-shaped P2RY12^+^ active microglia (Figure 7B). In contrast, RS102895 was unable to abate LPS-induced activation of microglia *in vitro*, suggest that this CCR2 antagonist lacks direct inhibitory effects on microglia (Figure S2D; n = 3 for each condition).

When two doses of 10 mg/kg RS102895 were given to the mouse neonates within 1 h post-LPS/HI, the mRNAs of *Tnf, Il1b, Il6, Il23, Ccl2, Nos2* and* Tspo* (inflammatory markers) were reduced at 24 h (Figure 7D; n > 3 in each group). This regimen of RS102895 treatment also attenuated the cerebral cortex and hippocampus degeneration at 7 d post-LPS/HI (Figure 7E-G). These results suggest acute and long-term benefits of the CCR2-inhibition treatment in neonatal LPS/HI brain injury. Finally, when evaluated by the novel objection recognition (NOR) test at 45 d post-LPS/HI, RS102895-treated mice spent more time with the novel (N) object than the familiar (F) object compared with vehicle-treated mice, despite similar total travel distance (Figure 7H; n = 9 for each group). These results suggest acute and long-term benefits of the CCR2-inhibition treatment in neonatal LPS/HI brain injury.

## Discussion

Monocytes belong to the mononuclear phagocyte system (MPS) and replenish many types of tissue-resident macrophages, but their relationship to microglia has been contentious 26, 27. The current prevailing view posits that the parenchymal microglia are derived wholly from the yolk sac progenitors and sustained by local self-renewal throughout adult life, while the blood-borne CCR2+ monocytes only replace microglia in defined host conditions 15, 16, 29, 30. Yet, two recent studies suggest that monocytes also convert to parenchymal microglia in development and after neonatal stroke 9, 31. The pathological functions of monocytes and whether or not they contribute to the microglial pool after neonatal HI injury are unclear 7. Elucidating these issues has implications for both basic science and clinical practice, since the influx of monocytes may be impeded by targeting the MCP1-CCR2 chemoattraction axis as shown in several past studies 17-19, 43-46. In this study, we used three fate-mapping and two CCR2-intervention methods to assess the plasticity and pathological roles of monocytes in neonatal LPS/HI brain injury. Herein we discuss our results in three areas.

### Monocytic influx through selected gateways in late embryonic brains

A previous study using CCR2^RFP/+^; CX3CR1^GFP/+^ embryos reported that RFP^+^ monocytes are confined to meningeal areas and absent in brain parenchyma, whereas GFP^+^ microglia reside in the neuroepithelium from E11.5 to P0 32. This study is often cited as evidence against monocytic contribution to microglia ontogeny. However, the classic literature emphasized that microglia cells enter cerebral tissue only through selected sites that are referred to as the “nests or fountains of microglia” 28, 33. These microglial fountains are characteristically located in a zone where the choroid plexus is in contact with brain parenchyma. Consistent with the literature, we found a mixture of GFP^+^, RFP^+^ and RFP^+^/GFP^+^-to-GFP^+^ cells in the choroid plexus between two primordial hippocampi in E17.5 R/G embryos (Figure 1). These results suggest that CCR2^+^ monocytes enter embryonic brain through specific gateways and alter their cell-surface markers afterwards. This transition of cell-surface markers resembles the development of CCR2^-^ CX3CR1^+^ Ly6C^lo^ patrolling monocytes from short-lived CCR2^+^ CX3CR1^-^ Ly6C^hi^ monocytes in the bone marrow 12. Moreover, monocytes were reported to undergo *in-situ* transition from a CCR2^hi^ CX3CR1^lo^ to CCR2^lo^ CX3CR1^hi^ state after sterile liver injury 22. The combination of past reports and our new results supports environment-dependent reprogramming of monocytes via transcriptional and/or epigenetic regulation 23.

Our results also add to the evidence for dual origins of the brain microglia, i.e. brain microglia originate from the yolk sac-derived progenitors and are subsequently added by the derivatives of hematopoietic monocytes 9, 29, 31. This scheme of layered ontogeny better matches the development of the other tissue-resident macrophages 27. Regarding the entry of fetal monocytes in embryonic brains, the MCP1-CCR2 chemoattractant axis is unlikely to play a key role, since CCR2^RFP/RFP^ mice do not suffer from obvious reduction of microglia 47. A more likely scenario is the presence of unidentified monocytic chemoattractant(s) in the microglial fountains that are characteristically “related to areas in which (axonal) tracts are being formed” 33. Further, since the blood vessels in choroid plexus lack tight-junctions and are in close proximity to embryonic brain parenchyma 48, these properties may assist the invasion of fetal monocytes. Future studies are needed to elucidate the chemoattractant signals and test whether the brain microglia of different origins have distinct functions and/or locations.

### Pathological functions by monocytes in neonatal HI brains injury

We showed in this study that the influx of monocytes declines rapidly after birth, which may relate to the growing separation of choroid plexus from mature brain parenchyma. However, the influx of monocytes reappeared following LPS-sensitized HI, and to a lesser degree, after pure-HI, in neonatal brains (Figure 2). We have also reported that prenatal/maternal immune activation enhances the influx of CCR2^+^ monocytes after neonatal HI injury 49. Together, these findings suggest the influx of monocytes after neonatal brain injury, particularly when compounded by perinatal inflammation 3.

Our results showed that invading monocytes first became macrophages (Mo-MΦ) and promoted the expression of pro-inflammatory cytokines after neonatal LPS/HI injury. While the resident microglia can also become macrophages (Mi-MΦ), they may have distinctive properties from Mo-MΦ. For example, we showed that MCP-1 is expressed by CX3CR1^+^ microglia, but not by CCR2^+^ monocytes. IL-1β is also expressed by a higher percentage of Mo-MΦ than Mi-MΦ. Moreover, when the infiltration of CCR2^+^ monocytes was hindered in LPS/HI, the expression of microglia-activation markers was markedly reduced (Figure 6 and Figure 7). This finding suggests that emigrant monocytes may instruct the formation of Mi-MΦ after neonatal brain injury.

Our results also support important functions of CCR2 for monocytic recruitment in inflammation-sensitized neonatal brain injury, as has been shown in a variety of neurologic insults 17-19, 43-46. Here we showed that genetic disruption or pharmacological blockade of CCR2, the obligate MCP-1 receptor, markedly reduced the influx of monocytes and the extent of LPS/HI brain damage, leading to better cognition functions. Of note, the CCR2-inhibitor used in our study (RS102859) only dissolves in organic solvent, but there are oral bioavailable CCR2 inhibitors that have been used in clinical trials for other diseases 43, 50, 51. These newer CCR2 inhibitors are more suited for clinical application in infants suffered from birth asphyxia or HIE, especially when compounded by chorioamnionitis, which carries a higher risk for cerebral palsy and poor responses to therapeutic hypothermia 1, 2.

### Ontogeny, environment, and heterogeneity of monocytic derivatives

In this study, we employed three fate-mapping methods to evaluate the outcomes of emigrant monocytes in neonatal brain injury. Each of these three methods has its unique strength and weakness. Specifically, CCR2^RFP/+^; CX3CR1^GFP/+^ mice are very efficient to survey the acute influx of CCR2^+^ monocytes after brain injury, but it is unable to distinguish Mi-MΦ (GFP^+^) from a subset of Mo-MΦ with certain microglia-like properties (CCR2^RFP-^ CX3CR1^GFP+^), as suggested by our results and previous studies 9, 22. Adoptive transfer of constitutive GFP^+^ monocytes is an alternative to parabiosis and bone marrow chimera (which are not applicable for studying neonatal injury) and provides constitutive labeling of monocytic derivatives, but its efficiency is low and variable. In contrast, inducible CCR2-CreER mice are particularly apt to fate-map monocytes during development and after neonatal brain injury 9. Moreover, by crossing with R26R-EGFP or R26R-EGFP/Rpl10A mice, the CCR2-CreER mice can be used to delineate the morphology and transcriptome of monocytic infiltrates, respectively.

Using CCR2-CreER mice, our results revealed three important features of monocytic derivates after neonatal LPS/HI injury. First, the lifespan of CCR2^+^ monocyte-derivatives is markedly expanded from a few days in the blood to 5 months in the brain 12. The increased longevity supports the idea of environment-dependent reprogramming of monocytes. Second, there is considerable heterogeneity of monocytic derivatives in the post-injury brains ranging from amoeboid macrophages to microglia-like ramified cells that express the microglia signatures genes (Figure 4 and Figure 5). This finding suggests that the ontogeny, environment, and heterogeneity are three critical factors in determining the phenotype of monocytic derivatives in the brain. Accordingly, bulk RNA-Sequencing of the transplanted monocytes is poised to show differences from the resident microglia, while single-cell RNA-Seq analysis is a better strategy to captures the full reprogramming potential of monocytic infiltrates 37, 52.

Last but not least, our results suggest that monocytic infiltrates may exert protracted deleterious effects after neonatal brain injury, and that cell morphology alone is unreliable to predict their functions. For example, TUNEL^+^ cell death remains detectable near monocytic derivatives one month after neonatal LPS/HI (Figure 5F). Further, the monocytic derivatives with microglia-like morphology can still express the pro-inflammatory TNFα (Figure 4P). These protracted deleterious effects suggest that monocytic infiltrates not only promote acute neonatal brain damage, but may also impair the development of immature brains 40, 49.

In conclusion, the results of our study suggest significant plasticity, pathological functions, and protracted harmful effects by monocytic infiltrates in inflammation-sensitized neonatal brain injury. Our results also suggest that targeting the chemokine receptor CCR2 may be a promising therapy of these neonatal insults.

## Supplementary Material

Supplementary figures.

## Abbreviations

AMCs: Amoeboid microglial cells
BBB: blood-brain-barrier
ChIP: chromatin immunoprecipitation
Ctx: cerebral cortex
CPx: choroid plexus
EAE: experimental autoimmune encephalitis
EC: external capsule
EdU: 5-ethynyl-2'-deoxyuridine
GE: ganglionic eminence
GW: gestational week
HI: hypoxic-ischemic
HIE: hypoxic-ischemic encephalopathy
Hip: hippocampus
IP: intraperitoneal
LPS: lipopolysaccharide
LV: lateral ventricle
MCP1: monocyte chemoattractant protein-1
Mi: microglia
Mo: monocytes
MΦ: macrophages
MPS: mononuclear phagocyte system
NOR: novel objection recognition
OCT: optimal cutting-temperature
R/G: CCR2^RFP/+^, CX3CR1^GFP/+^
St: striatum
Syn: synaptotagmin
TRAP: translating ribosome affinity purification
TUNEL: terminal deoxynucleotidyl transferase dUTP nick end labeling
UN: uninjured
