# Supplementary material for: Monocytes promote acute neuroinflammation and become pathological microglia in neonatal hypoxic-ischemic brain injury
Source: Theranostics. 2022 Jan 1;12(2):512–29. doi: 10.7150/thno.64033 (PMC8692901; doi:10.7150/thno.64033)
Supplement: Supplementary file 1 — Supplementary figures. [file thnov12p0512s1.pdf]

Figure S1

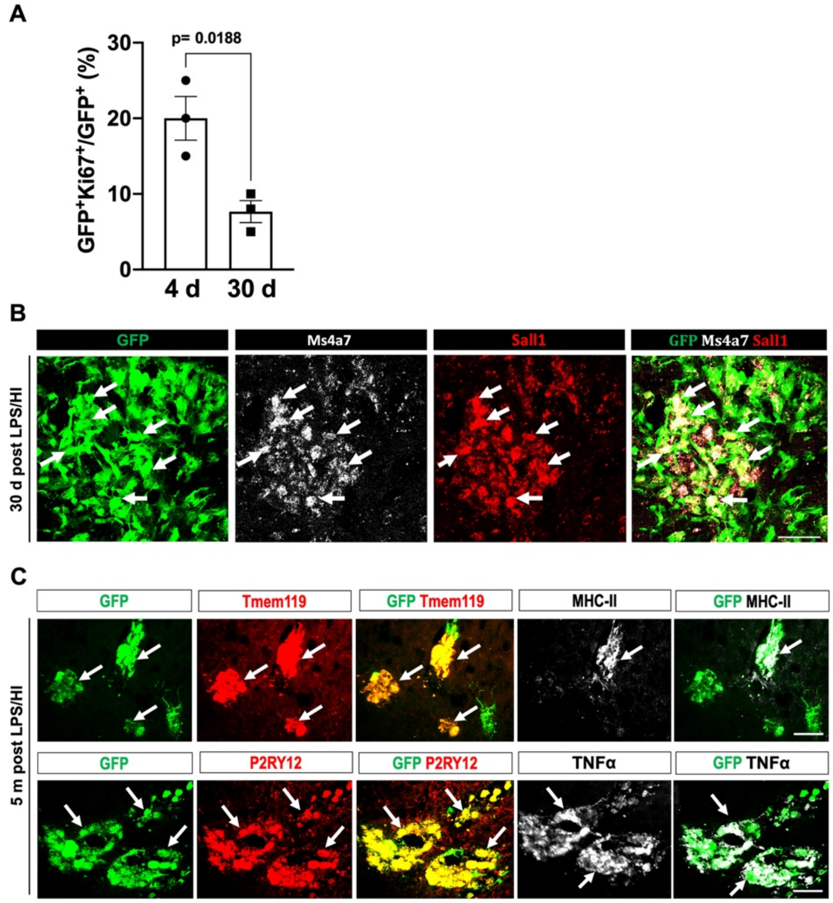

Figure S1

(A) Quantification of GFP$^+$Ki67$^+$ cells in the brains of CCR2-CreER; R26R-GFP mice at 4 d and 30 d after neonatal stroke (n = 3 of both genders). **(B)** Co-expression of *Sall1* and *Ms4a7* on CCR2$^+$ monocyte derivatives at 30 d post-LPS/HI. CCR2-CreER; R26R-GFP mice were subjected to tamoxifen injection at P8 and P9, followed by LPS/HI injury at P10. Brains were harvested at 30 d after the injury. RNA scope images show that GFP$^+$ monocyte derivatives express both *Ms4a7* (white) and *Sall1* (red) mRNA (arrows) (n = 5). Scale bar: 50 μm. **(C)** At 5 m post-LPS/HI, clumps of GFP$^+$ derivatives expressed MHC-II, TNFα, and the microglial markers (Tmem119 and P2RY12).

Figure S2

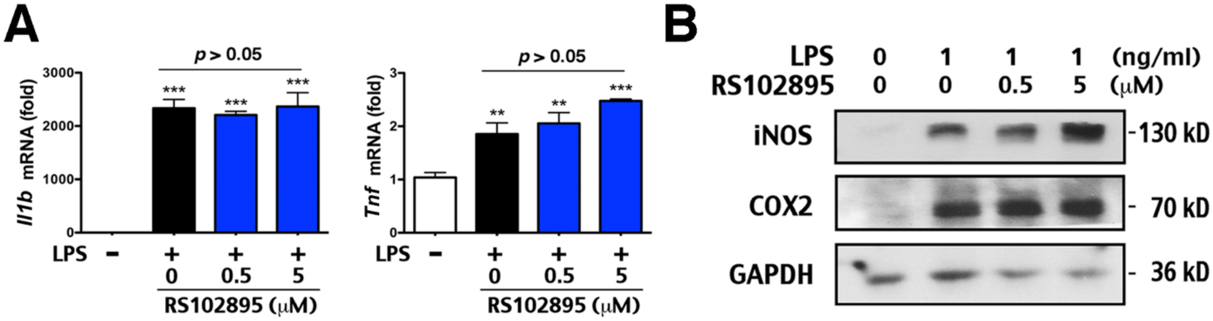

Figure S2

Incubation of immortalized microglia SM826 cells with 1 ng/ml LPS markedly elevated the *Il1b* and *Tnf* mRNAs (**A**) plus iNOS and COX2 protein (**B**) 24 h later. The addition of 0.5 or 5 μM RS102895 failed to abate these responses, suggesting lack of direct inhibitory effects on microglia. N = 3 for each group.
